# Supplementary material for: Digital exposure notification tools: A global landscape analysis
Source: PLOS Digit Health. 2023 Sep 1;2(9):e0000287. doi: 10.1371/journal.pdig.0000287 (PMC10473497; doi:10.1371/journal.pdig.0000287)
Supplement: Attachment — Submitted filename: PLOS DH Response to Reviewers 032823.docx [file pdig.0000287.s007.docx]

**Response to Reviewers**

Thank you for your thoughtful comments and feedback. Below we provide our response to each comment.

1. Please send a completed 'Competing Interests' statement, including any COIs declared by your co-authors. If you have no competing interests to declare, please state "The authors have declared that no competing interests exist". Otherwise please declare all competing interests beginning with the statement "I have read the journal's policy and the authors of this manuscript have the following competing interests:"

RESPONSE: "The authors have declared that no competing interests exist".

2. . Please amend your detailed Financial Disclosure statement. This is published with the article. It must therefore be completed in full sentences and contain the exact wording you wish to be published.

RESPONSE: We have amended the text with the following

“The authors disclosed receipt of the following financial support for the research, authorship, and/or publication of this article: the authors received funding from the California Department of Public Health, agreement 20-10777. The funders had no role in study design, data collection and analysis, decision to publish, or preparation of the manuscript. Authors EAS, CN, RK and MM did receive partial salary support from the funders.”

3. We notice that your revision was submitted on Mar 6, 2023, but the manuscript file in your submission's file inventory was uploaded on May 9, 2022. Please upload the latest version of your revised manuscript as the main article file, with the item type 'Manuscript,' ensuring that it does not contain any tracked changes or highlighting. This will be used in the production process if your manuscript is accepted.

RESPONSE: We have uploaded a new manuscript with the current days date.

4. We do not publish any copyright or trademark symbols that usually accompany proprietary names, eg  ©, ®, ™  (e.g. next to drug or reagent names). Please remove all instances of trademark/copyright symbols throughout the text, including ® on page 27.

RESPONSE: Removed

5. Figure 8: please (a) provide a direct link to the base layer of the map (i.e., the country or region border shape) and ensure this is also included in the figure legend; and (b) provide a link to the terms of use / license information for the base layer image or shapefile. We cannot publish proprietary or copyrighted maps (e.g. Google Maps, Mapquest) and the terms of use for your map base layer must be compatible with our CC-BY 4.0 license.

Note: if you created the map in a software program like R or ArcGIS, please locate and indicate the source of the basemap shapefile onto which data has been plotted.

If your map was obtained from a copyrighted source please amend the figure so that the base map used is from an openly available source. Alternatively, please provide explicit written permission from the copyright holder granting you the right to publish the material under our CC-BY 4.0 license.

Please note that the following CC BY licenses are compatible with PLOS license: CC BY 4.0, CC BY 2.0 and CC BY 3.0, meanwhile such licenses as CC BY-ND 3.0 and others are not compatible due to additional restrictions.

RESPONSE: We have revised the table and present the license and link below.

**A) The global map was created using Tableau software which uses OpenStreetmap. OpenStreetMap**[**^®^**](https://urldefense.com/v3/__https:/www.openstreetmap.org/copyright/*trademarks__;Iw!!LLK065n_VXAQ!lPzqL8nzlslyVwxaoHEgjMY3jDeD1Rd15UtRT0V_rzIPzzK_81wh4k_8h6ZuNPA9U1jXXdlv0lRBY96oOB6X$) **is *open data*, licensed under the** [**Open Data Commons Open Database License**](https://urldefense.com/v3/__https:/opendatacommons.org/licenses/odbl/__;!!LLK065n_VXAQ!lPzqL8nzlslyVwxaoHEgjMY3jDeD1Rd15UtRT0V_rzIPzzK_81wh4k_8h6ZuNPA9U1jXXdlv0lRBYyA6cOfw$) **(ODbL) by the** [**OpenStreetMap Foundation**](https://urldefense.com/v3/__https:/osmfoundation.org/__;!!LLK065n_VXAQ!lPzqL8nzlslyVwxaoHEgjMY3jDeD1Rd15UtRT0V_rzIPzzK_81wh4k_8h6ZuNPA9U1jXXdlv0lRBY-gXEUy7$) **(OSMF).** [**Link to Copyright License**](https://urldefense.com/v3/__https:/www.openstreetmap.org/copyright__;!!LLK065n_VXAQ!lPzqL8nzlslyVwxaoHEgjMY3jDeD1Rd15UtRT0V_rzIPzzK_81wh4k_8h6ZuNPA9U1jXXdlv0lRBY36BDq7o$)**.**

**B) Link is provided for map in the legend associated with the map :** [**https://opendatacommons.org/licenses/odbl/**](https://opendatacommons.org/licenses/odbl/)
